# Feature Selection and Prediction of Pediatric *Tuina* in Attention Deficit/Hyperactivity Disorder Management: A Machine Learning Approach Based on Parent-Reported Children’s Constitution

**DOI:** 10.3390/bioengineering12101012

**Published:** 2025-09-23

**Authors:** Shu-Cheng Chen, Guo-Tao Wu, Han Li, Xuan Zhang, Zi-Han Li, Pong-Ming Wong, Le-Fei Han, Jing Qin, Kwai-Ching Lo, Wing-Fai Yeung, Ge Ren

**Affiliations:** 1 Centre for Smart Health, School of Nursing, The Hong Kong Polytechnic University, Hung Hom, Hong Kong, China; cara-sc.chen@polyu.edu.hk (S.-C.C.); harry.qin@polyu.edu.hk (J.Q.); 2 Psychology Department, Southwest University, Chongqing 402460, China; 3 School of Nursing, The Hong Kong Polytechnic University, Hung Hom, Hong Kong, Chinajerry-wf.yeung@polyu.edu.hk (W.-F.Y.); 4 Department of Pediatric *Tuina*, Affiliated Hospital of Shandong University of Traditional Chinese Medicine, Jinan 250355, China; 5 Department of Health Technology and Informatics, The Hong Kong Polytechnic University, Hung Hom, Hong Kong, China; 6 School of Chinese Medicine, The University of Hong Kong, Hong Kong, China; 7 School of Global Health, Chinese Center for Tropical Diseases Research, Shanghai Jiao Tong University School of Medicine, Shanghai 200025, China; 8 Research Center for Chinese Medicine Innovation, The Hong Kong Polytechnic University, Hung Hom, Hong Kong, China; 9 Research Institute for Smart Ageing, The Hong Kong Polytechnic University, Hung Hom, Hong Kong, China

**Keywords:** ADHD, machine learning, feature selection, TCM pattern identification, pediatric *tuina*

## Abstract

***Background***: Attention Deficit/Hyperactivity Disorder (ADHD) is a common neurodevelopmental disorder in children. Pediatric *tuina*, a traditional Chinese medicine (TCM) intervention, has shown potential in managing ADHD symptoms. Integrating machine learning (ML) into pediatric *tuina* could refine treatment personalization, allowing for a more feasible and better parent-administered use. ***Methods***: We employed an ML-based model to analyze parent-reported constitutional features from 1005 children diagnosed with ADHD to predict individualized pediatric *tuina* treatments. This study focused on feature selection and the application of several ML models, including Support Vector Machines (SVM), Logistic Regression (LR), Multilayer Perceptron (MLP), and Random Forest (RF). The key task involved identifying the most relevant features for effective TCM pattern identification and diagnosis, which would guide personalized treatment strategies. ***Results***: The ML models displayed strong predictive performance, with the MLP model achieving the highest Area Under the Curve (AUC) of 0.90 and an accuracy (ACC) of 0.74. Seven features were selected five times in cross-validation. This facilitated a more targeted and effective pediatric *tuina* application tailored to individual constitution. ***Conclusion***: This study developed an ML-based approach to enhance ADHD management in children using pediatric *tuina*, informed by a parent-reported questionnaire. It identified seven key features for TCM pattern identification and personalized treatment strategies. MLP achieved the highest AUC and ACC.

## 1. Introduction

### 1.1. Attention Deficit/Hyperactivity Disorder in Children

Attention Deficit/Hyperactivity Disorder (ADHD) is a neurodevelopmental disorder characterized by pervasive patterns of inattention, hyperactivity, and impulsivity that significantly interfere with daily functioning and development [1]. ADHD affects approximately 5–10% of children worldwide, making it one of the most common psychiatric disorders in pediatric populations [2]. The disorder typically manifests in early childhood and can persist into adolescence and adulthood [3]. Children with ADHD often struggle with maintaining attention, following instructions, and controlling impulses [4], which can lead to academic difficulties [5], strained peer relationships [6], and low self-esteem [7]. Conventional interventions for ADHD primarily include pharmacotherapy [8], such as stimulant medications (e.g., methylphenidate and amphetamines), and behavioral therapies that aim to modify disruptive behaviors and improve organizational skills [9]. However, these treatments may not be effective for all children and can be associated with undesirable side effects or requiring high parental involvement [10]. As a result, there is a growing interest in complementary and alternative interventions, such as dietary modifications [11], neurofeedback [12], mindfulness training [13], acupuncture [14], or massage [15]. Complementary interventions are important as they offer holistic approaches that can address multiple dimensions of ADHD, potentially enhancing symptom management and overall quality of life for children and their families [16]. By integrating these approaches with conventional treatments, healthcare providers can offer more personalized and effective care strategies, aligning with the principles of predictive, preventive, and personalized medicine [17].

### 1.2. Pediatric Tuina for ADHD Management

Pediatric *tuina*, also known as pediatric *anmo* or Traditional Chinese Medicine (TCM) pediatric massage, is a specialized modality of therapeutic massage rooted in TCM, designed specifically for children [18]. Unlike adult *tuina*, pediatric *tuina* has its unique manipulation techniques and specific acupoints. The shape of acupoints (e.g., line, circle, and area) and techniques are closely related to the meridian pathways and body’s anatomical structures. The techniques involve a series of manipulations, including linear rubbing, circular rubbing, pushing along specific lines, and point pressing, which are performed with different frequencies, directions, and intensities on specific acupoints or meridians to stimulate physiological functions and promote health [19]. Pediatric *tuina* is commonly employed to address a range of childhood ailments, including digestive disorders, respiratory conditions, musculoskeletal problems, and so forth [20]. In the context of ADHD management, pediatric *tuina* aims to harmonize the body’s energy flow, enhance mental focus, and reduce hyperactivity by stimulating acupoints related to the nervous system and brain function [15,21]. The underlying mechanism might be related to the modulation of neurotransmitters and improvement of cerebral blood flow, thereby exerting a calming effect and enhancing cognitive functions [22,23]. In TCM theory, a pattern represents a set of characteristic features or symptoms that reflect a patient’s overall health condition. For ADHD management, TCM practitioners commonly differentiate children into several patterns, including dual deficiency of the lung–spleen pattern, liver depression and spleen deficiency pattern, liver ascendant hyperactivity and spleen deficiency pattern, effulgent heart–liver fire pattern, phlegm-fire harassing the heart pattern, and liver–kidney yin deficiency pattern. Each pattern corresponds to specific therapeutic approaches. Findings from previous studies on pediatric *tuina* for ADHD suggest that pediatric *tuina* has beneficial effects on improving ADHD symptoms on children and could serve as a valuable complementary therapy. For instance, a pilot randomized controlled trial (RCT) demonstrated the feasibility and beneficial effects of parent-administered pediatric *tuina* on reducing core symptoms in preschool children with ADHD [24]. Furthermore, the qualitative findings from focus group interviews highlighted the perceived benefits of pediatric *tuina* on children’s sleep quality, appetite, and parent–child relationships, although some parents noted limited improvements in inattention symptoms [25].

### 1.3. The Potential of Machine Learning in Pediatric Tuina for ADHD

Machine learning (ML) is a branch of artificial intelligence that focuses on developing algorithms capable of learning from and making predictions based on data. In TCM, ML shows promise in improving personalized treatment strategies. By developing robust prediction models, ML can optimize treatment protocols tailored to individual patient characteristics, thereby enhancing therapeutic outcomes and patient care. Several previous studies have successfully applied ML to similar tasks in the TCM area, demonstrating its potential to improve diagnostic accuracy (ACC) and treatment outcomes [26,27,28,29,30,31]. A study demonstrated that a multi-feature TCM constitution identification model integrating facial complexion, body shape features, and deep features could achieve an accuracy of 0.842, providing strong evidence for the effectiveness of comprehensive feature fusion approaches in TCM constitution classification [30]. A study developed an ML-assisted rapid determination methodology for TCM constitution based on the Constitution in Chinese Medicine Questionnaire, achieving classification accuracies of 0.819–0.936, demonstrating that a subset of core items can effectively predict body constitution [31]. Our team previously conducted two pilot RCTs focused on parent-administered pediatric *tuina* for ADHD in children (NCT04237259/NCT06007742). In these studies, TCM practitioners provided individualized pediatric *tuina* prescriptions based on pattern identification and diagnosis for each child. Pattern differentiation serves as the cornerstone of TCM treatment planning. In pediatric *tuina* for ADHD, different patterns guide practitioners to select specific acupoints and manipulation techniques. This individualization is crucial as it directly affects treatment outcomes—appropriate pattern-based treatment typically leads to better therapeutic effects and fewer adverse events compared to standardized approaches. Using inappropriate treatments not matching the pattern may result in reduced effectiveness or even adverse reactions. Parents were trained by TCM practitioners on the required manipulations and performed these at home over an 8-week period. The diagnoses were based on parent-reported children’s constitutional questionnaires. These studies confirmed the feasibility, acceptability, and preliminary efficacy of this approach. Conducted in mainland China and Hong Kong, the studies included both preschool and school-aged children, demonstrating wide applicability. Given the high prevalence of ADHD, refining and expanding these methods using ML could make this intervention accessible to more people, assist novice TCM practitioners in pattern identification and diagnosis, and help parents better understand their children’s constitution. Therefore, we conducted this study.

### 1.4. Objectives of This Study

The primary objective of this study was to develop an ML-based approach for feature analysis and pediatric *tuina* prescriptions prediction in the management of ADHD based on parent-reported child constitutional information. Specifically, this study aimed to (1) select the most relevant features for effective TCM pattern identification and diagnosis; (2) predict the TCM pattern type for each child with ADHD to recommend individualized parent-administered pediatric *tuina* prescriptions; and (3) identify the most suitable ML model for the task.

## 2. Methods

**Figure 1:**
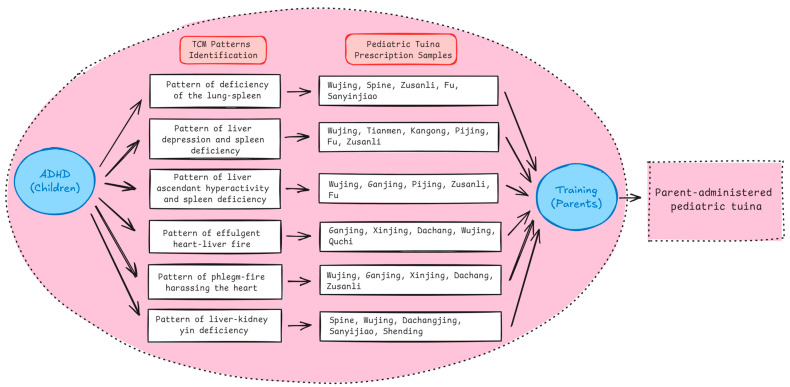
TCM pattern identification and prescription formulation of parent-administered pediatric *tuina* for children with ADHD.

This study was an extension of a pilot RCT focused on parent-administered pediatric *tuina* for managing ADHD in school-aged children in Hong Kong (ClinicalTrials.gov: NCT06007742). The pilot RCT, which involved 61 parent–child pairs, used parent-reported questionnaires to collect information about the children’s constitution for TCM pattern identification. TCM practitioners performed TCM pattern identification for the children based on their constitutional information collected and formulated individualized pediatric *tuina* prescriptions for the parents to deliver on their children at home. Figure 1 shows the TCM patterns of ADHD and several prescription samples for each ADHD TCM pattern. The diagnosis and treatment framework were refined by our research team based on the pediatric clinical practice guideline of TCM for children with ADHD [29]. Findings of the study demonstrated that this method for children’s TCM pattern identification and parent-administered pediatric *tuina* prescription formulation were feasible. The current work is an application of the parent-reported questionnaire of children’s constitutional characteristics to develop an ML-based approach for selecting features and predicting pediatric *tuina* prescriptions for ADHD management.

### 2.1. Data Source

Data were collected from November 2023 to June 2024. The data utilized in this study were derived from a cross-sectional study in China using *Qualtrics (Provo, UT, USA) and Wenjuanxing (Changsha, China)*, both of which are widely used online survey platforms. The participants in this study were parents of children diagnosed with ADHD. A total of 1005 parents filled in the questionnaire of children’s constitutional characteristics. All participants met the following inclusion criteria: (1) were parents of children aged between 4 and 15 years, (2) their children had a clinical diagnosis of ADHD based on DSM-5 criteria [1], and (3) their children had no significant neurological, psychiatric, or medical conditions other than ADHD. Informed consent was obtained from the parents.

### 2.2. Data Processing

The dataset included 1005 questionnaires, each with 71 questions and corresponding labels. We labeled each child’s constitution as a TCM pattern based on the 71 features, which include 70 items derived from the parent-reported Child’s Constitutional Questionnaire and the child’s age as a demographic feature. This questionnaire, detailed in the document “Classification and Determination of Children’s Constitution in TCM,” was specifically designed to assess various aspects of children’s physical and psychological health according to TCM principles. The parent-reported section encompasses a range of items such as energy levels, appetite, sleep quality, bowel movements, spontaneous sweating, frequent colds, digestive issues, and emotional stability. Supplementary S1 lists these features in detail. Different options within the questionnaire were numerically coded; for example, Likert scale responses were assigned different numerical values, while “yes” or “no” options were represented by 0 or 1.

Four experienced TCM practitioners collaborated to diagnose and identify TCM patterns for each child. The lead TCM practitioner, KCL, who possesses over a decade of clinical experience in pediatric tuina, developed guidelines for identifying ADHD-related TCM patterns. Additionally, she supervised and verified the diagnostic outcomes provided by the other three practitioners. All children were assigned individualized TCM patterns for ADHD, including dual deficiency of the lung–spleen pattern, liver depression and spleen deficiency pattern, liver ascendant hyperactivity and spleen deficiency pattern, effulgent heart–liver fire pattern, phlegm-fire harassing the heart pattern, and liver–kidney yin deficiency pattern. The TCM patterns for ADHD referred to the TCM guidance for pediatric ADHD [32,33] and were adjusted by the TCM practitioners based on their clinical and research experience. Supplementary S2 presents the TCM pattern identification approaches and corresponding pediatric tuina prescriptions for ADHD in children. For the purposes of our ML model, we encoded the patterns as follows: dual deficiency of the lung–spleen was labeled as 0, liver depression and spleen deficiency as 1, and liver ascendant hyperactivity and spleen deficiency as 2. Due to the low prevalence of the remaining three patterns in clinical practice, we combined them into a single category labeled as 3. The three less common TCM patterns were combined due to their low frequencies in our dataset, as separate analysis could potentially compromise model reliability due to data sparsity.

### 2.3. Machine Learning Workflow

The task was implemented in two phases: the feature selection phase and the TCM pattern identification phase. During the feature selection phase, critical features were differentiated within the entire dataset. In the TCM pattern identification phase, four ML models were applied. All algorithms were implemented in Python 3.12 (Python Software Foundation, Wilmington, DE, USA).

#### 2.3.1. Feature Selection Phase

We initially conducted a feature correlation analysis employing correlation coefficient methodology to assess the redundancy among the features. Then the feature selection phase was implemented using a robust approach that involved a linear regression classifier with five-fold cross-validation. The dataset was divided into five subsets. In each fold, one subset was used for validation while the remaining four subsets were used for training. This process was repeated five times, ensuring that each subset was used for validation exactly once. This method helps in providing reliable performance estimates and reducing overfitting risk. In each fold, the Logistic Regression classifier using the log loss (Formula (1)) penalizes incorrect predictions with greater severity as the predicted probability diverges from the actual label. It ranked the features based on their contribution to the Area Under the Curve (AUC) values. This ranking process allowed us to identify which features had the most significant impact on the model’s predictive ability. The number of features considered ranged from 1 to 71. After identifying the critical features, these selected features were used to train and test the ML models in the subsequent TCM pattern identification phase.

(1) $$Log\;Loss=-\frac{1}{N}\sum_{i=1}^{N}\sum_{k=1}^{K}y_{ik}\log\left(p_{ik}\right)$$

where *K* is the number of classes, $y_{ik}$ is a binary indicator (0 or 1) that signifies whether class label *k* is the correct classification for sample *i*, and $p_{ik}$ is the predicted probability that sample *i* belongs to class *k*. A lower log loss value indicates better model performance, as it reflects more accurate predictions.

#### 2.3.2. TCM Pattern Identification Phase

In the TCM pattern identification phase, multiple ML models were constructed and evaluated to identify the most effective model for TCM pattern classification in children with ADHD. The models applied included Support Vector Machines (SVM), Logistic Regression (LR), Multilayer Perceptron (MLP), and Random Forest (RF). SVM is particularly effective for high-dimensional spaces and is commonly used for classification tasks, constructing a hyperplane that best separates the classes in the feature space [34]. LR is a widely used statistical model for binary classification tasks, providing interpretable feature importance, making it easier to understand the influence of each feature on the prediction [35]. MLP, a type of artificial neural network, is capable of capturing complex, non-linear patterns in the data through multiple layers of nodes, each using a non-linear activation function [36]. RF, an ensemble learning method, combines multiple decision trees to improve predictive accuracy (ACC) by averaging the predictions from various trees, which reduces overfitting and enhances generalization [37].

The dataset was divided into 80% for training and cross-validation and 20% for testing. The training subset was used to train the models, while the validation subset was employed for hyperparameter tuning to avoid overfitting. The final model evaluation was performed on the test subset to assess its predictive performance. Evaluation metrics included the AUC and ACC for both the training and testing datasets. These metrics provided a comprehensive assessment of each model’s performance, ensuring that the selected model not only had high AUC scores but also balanced ACC between the training and testing datasets. The model demonstrating the best overall performance across these metrics was selected for TCM pattern identification.

In our study, the classifiers were standard ML models that required only limited hyperparameter tuning, with typically one or two key parameters set for each. Specifically, the MLP was configured with a hidden layer size of three layers containing 64 units each, together with an adaptive learning rate. The support vector machine (SVM) was parameterized by the kernel width (γ), which was set to auto under the RBF kernel. The Random Forest (RF) was constructed with 70 trees (n_estimators = 70) without a predefined maximum depth (max_depth = None). For LR, the main hyperparameter was the regularization strength, optimized under the L-BFGS solver with a maximum of 1000 iterations (max_iter = 1000). These parameter values were first adopted based on established practices and prior experience and then further refined through empirical evaluation on our discovery cohort. To ensure robustness, we conducted sensitivity analyses of the key hyperparameters (e.g., hidden layer size for MLP, number of trees for RF, regularization strength for LR, and kernel width for SVM). The reported settings consistently yielded the best performance for each classifier on our discovery dataset and were therefore used for subsequent comparative analyses.

### 2.4. Statistical Analysis

To ensure the robustness and reliability of the ML models, statistical analysis focused on evaluating the performance metrics through cross-validation. The primary evaluation metrics included the AUC (Formula (2)) and ACC for both the training and testing datasets. *ACC* measures the proportion of correctly predicted samples out of all examined cases. Mathematically, it is defined as:

(2) $$ACC=\frac{PC+NC}{PC+NC+PE+NE}$$

where *PC* is the number of samples correctly predicted as a specific TCM pattern, *NC* is the number of samples correctly predicted as not belonging to that TCM pattern, *PE* represents the number of samples incorrectly predicted as that TCM pattern, and *NE* represents the number of samples incorrectly predicted as not belonging to that TCM pattern. Confidence intervals for these metrics were calculated to assess the precision of the performance estimates. Hyperparameter tuning was validated using the validation subset, ensuring that the tuning process effectively mitigated overfitting and enhanced the models’ generalization capabilities. This rigorous statistical analysis framework provided a reliable basis for identifying TCM patterns in children with ADHD, ensuring that the selected model demonstrated statistically sound performance.

## 3. Results

### 3.1. Patient Characteristics

**Figure 2:**
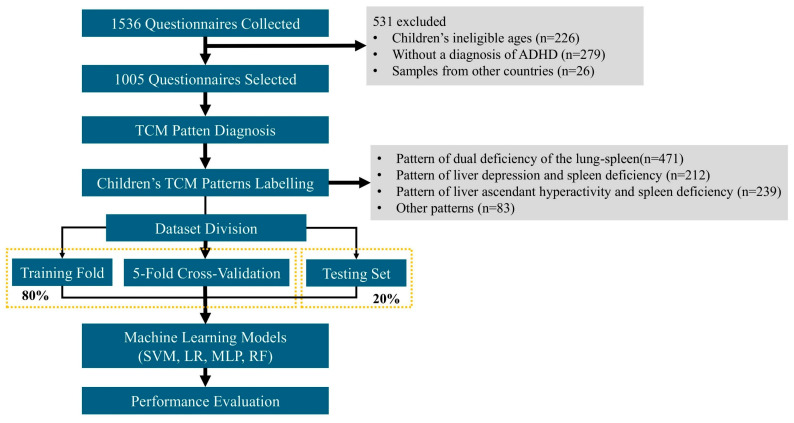
The flowchart of this study.

A detailed flowchart of the study is shown in Figure 2. A total of 1536 parents of children with ADHD completed and submitted the questionnaire. The questionnaires were collected from 32 multiple provincial administrative regions in China. According to the inclusion criteria, 531 questionnaires were excluded as follows: 226 questionnaires with children of ineligible age (<4 years or >15 years), 279 questionnaires with children not diagnosed with ADHD, and 26 questionnaires with children from other countries. Finally, 1005 eligible patients were included in the subsequent analysis. The selected participants had a median age of 10.0 [interquartile range (IQR) 9.0–11.0] years, with a female proportion of 17.3%. The number of participants diagnosed with a pattern of dual deficiency of the lung–spleen (labeled as 0), liver depression and spleen deficiency (labeled as 1), liver ascendant hyperactivity and spleen deficiency (labeled as 2), and others (labeled as 3) were 471, 212, 239, and 83, respectively. Table 1 shows the socio-demographic characteristics of parents and children labeled as different TCM patterns (*n* = 1005).

### 3.2. Feature Correlation Analysis

**Figure 3:**
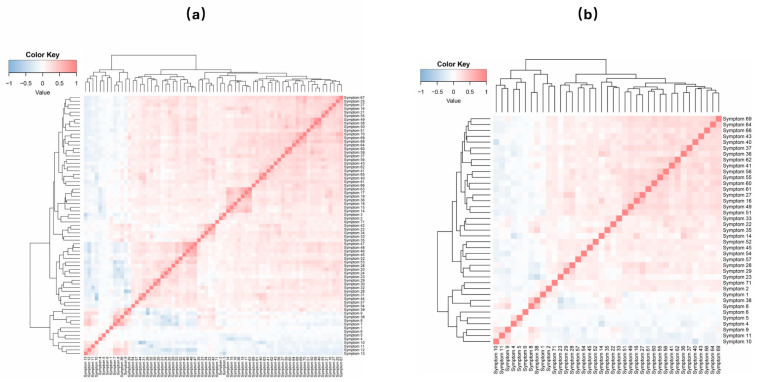
The correlation analysis between all features (**a**) and high correlation features (**b**). **Remarks**: The color scale ranges from −1.0 (dark blue, strong negative correlation) to +1.0 (dark red, strong positive correlation). Features with absolute correlation coefficients |r| > 0.5 are considered highly correlated. For detailed descriptions of all features (1–71), please refer to Supplementary S1.

To assess the redundancy of the questionnaire, we undertook an initial screening of the 71 features. The heatmap depicted in Figure 3a reveals a prevalent pattern of positive correlations among the features, with a notable minority exhibiting negative correlations (see Supplementary S3a and S3b). Specifically, several features demonstrate high correlation coefficients, with an absolute value above 0.5. For example, feature 10 (“The child falls asleep quickly and sleeps soundly”) and feature 44 (“The child takes a long time to fall asleep”) have a strong negative correlation of 0.72, whereas feature 50 (“The child feels like there’s something stuck in their throat”) and feature 60 (“The child easily has a lot of phlegm when coughing”) are positively correlated at 0.58. The strong correlation (where the absolute correlation exceeded 0.5) features are listed in Figure 3b. This process led to the elimination of 32 features, narrowing down the list from 71 to 39 features (see Supplementary S3c and S3d). These remaining features showed low intercorrelations, primarily weak positive and negative correlations.

### 3.3. Selection of the Features

**Figure 4:**
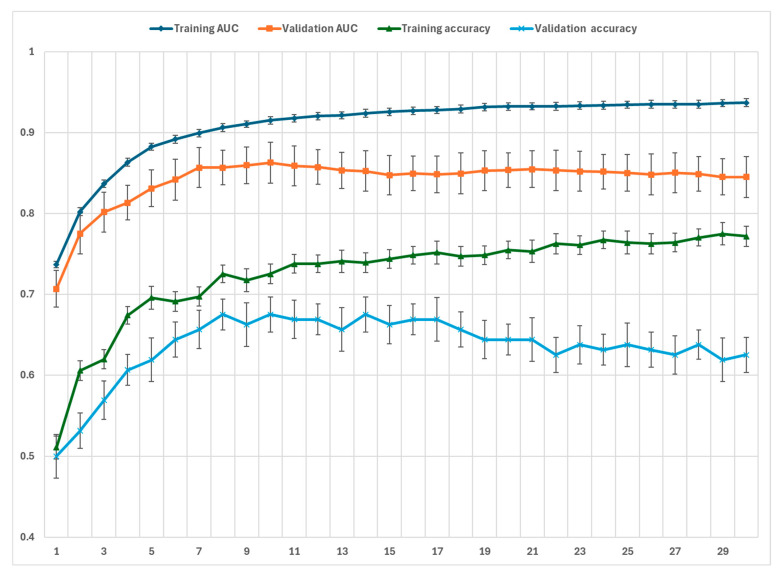
The training and validation performance as the number of features varies.

**Figure 5:**
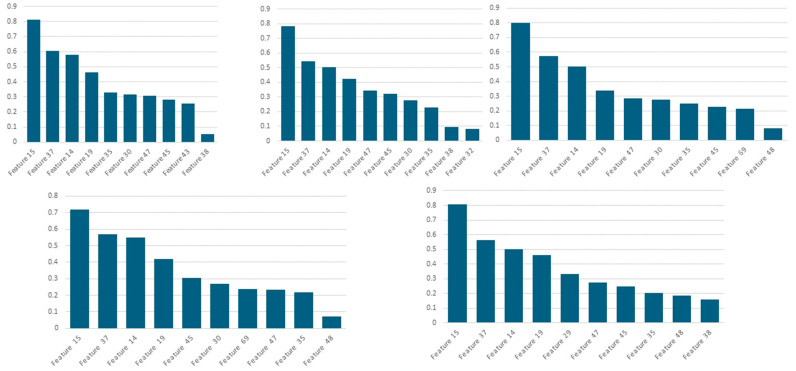
Importance maps of five-fold cross-validation. **Remarks**: [**Allergic symptoms**] feature 14: The child experiences itching after contact with or consuming allergens (such as certain foods, pollen, dust, pets, etc.); feature 15: The child is prone to allergic diseases, such as allergic rhinitis or cough-variant asthma, or tends to sneeze, have a runny nose, nasal congestion, or cough in situations like seasonal changes, temperature fluctuations, or exposure to pollen or furry animals, or in environments with potential allergens like renovations; feature 19: The child had chronic diarrhea or eczema when they were younger. [**Emotional/Psychological symptoms**] Feature 47: The child easily feels anxious and overthinks things; feature 48: The child remains in a low mood for a long time after setbacks; feature 45: The child is sensitive and thoughtful, caring a lot about others’ opinions; feature 35: The child has a quick temper; feature 38: The child is highly energetic and very active. [**Digestive symptoms**] Feature 69: The child tends to overeat and has indigestion; feature 32: The child has dry stool. [**Physical symptoms**] Feature 29: The child is afraid of the cold; feature 30: The child has cold hands and feet; feature 37: The child easily develops mouth ulcers or sore throats; feature 43: The child has a lot of eye discharge upon waking.

To select the most informative questions, we conducted a series of five-fold CV experiments using different numbers of input features, ranging from 1 to 30. As shown in Figure 4, when the model was provided with 10 input features, it achieved the highest average performance on the validation sets in terms of both AUC and ACC. Specifically, the key performance metrics are as follows: training AUC of 0.913; average validation AUC of 0.886; training ACC of 0.731; and average validation ACC of 0.702. Figure 5 presents the importance of each selected feature, arranged based on their selection frequency and importance values in the five-fold cross-validation. Among the five-fold CV, seven features were selected for five times as follows: feature 15, 14, 19, 37, 35, 47, and 45. Here, feature 15, 14, 37, and 19 have an importance value over 0.4.

The items from the questionnaire corresponding with the selected seven features are outlined as follows: (1) feature 15—“The child is prone to allergic diseases, such as allergic rhinitis or cough-variant asthma, or tends to sneeze, have a runny nose, nasal congestion, or cough in situations like seasonal changes, temperature fluctuations, or exposure to pollen or furry animals, or in environments with potential allergens like renovations”; (2) feature 14—“The child experiences itching after contact with or consuming allergens (such as certain foods, pollen, dust, pets, etc.)”; (3) feature 19—“The child had chronic diarrhea or eczema when they were younger”; (4) feature 37—“The child easily develops mouth ulcers or sore throats”; (5) feature 35—“The child has a quick temper”; (6) feature 47—“The child easily feels anxious and overthinks things”; and (7) feature 45—“The child is sensitive and thoughtful, caring a lot about others’ opinions”.

### 3.4. Predictive Performance of ML Models

**Figure 6:**
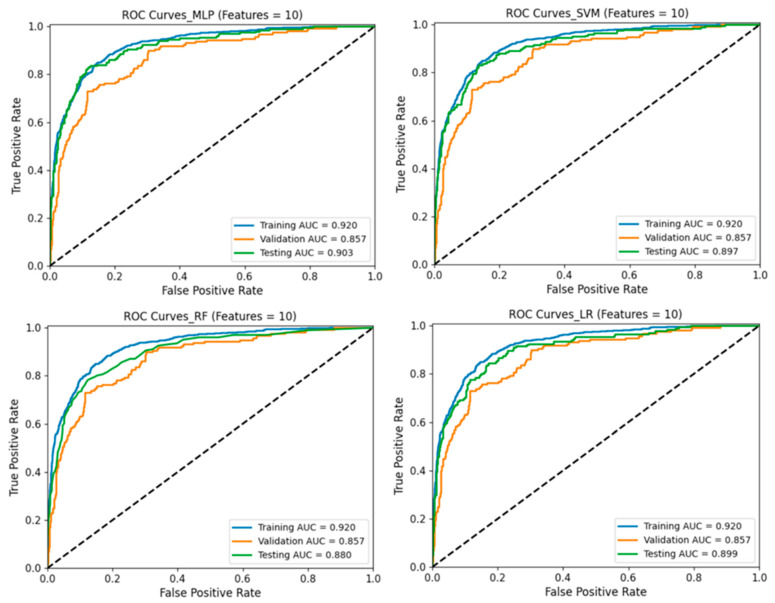
ROC curves for training, validation, and testing datasets (features = 10).

**Figure 7:**
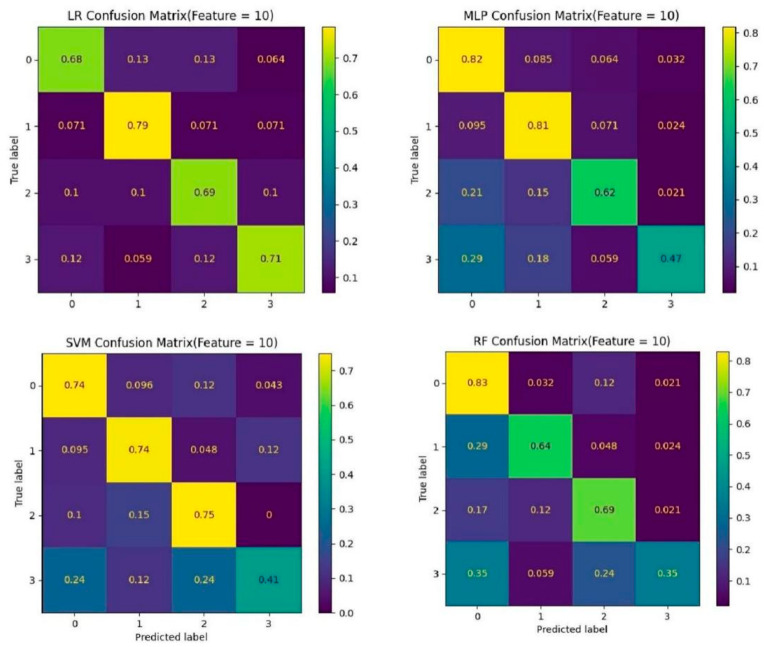
**Confusion metrics for the testing dataset. Remarks**: type 0: pattern of dual deficiency of the lung–spleen; type 1: pattern of liver depression and spleen deficiency; type 2: pattern of liver ascendant hyperactivity and spleen deficiency; type 3: other patterns.

**Figure 8:**
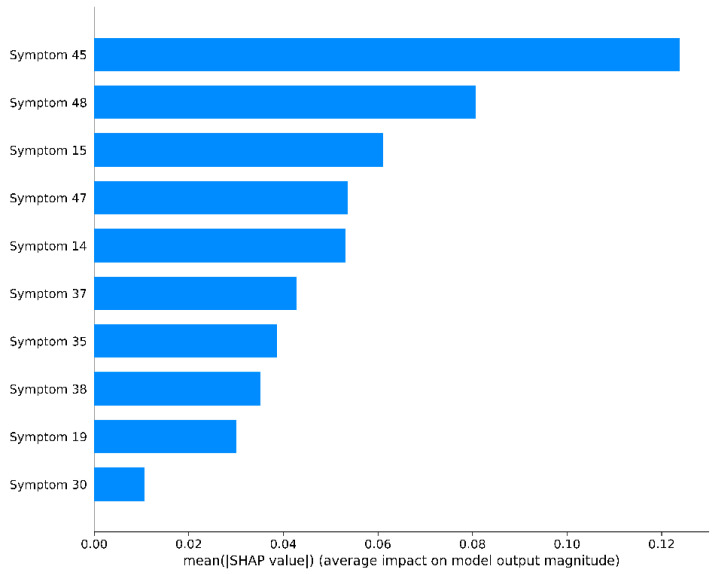
SHAP bar plot for the final MLP classifier.

In the testing cohort, the results showed that the MLP algorithm achieved the highest AUC of 0.90 (Figure 6) and the highest ACC of 0.74. All the four ML algorithms achieved AUC > 0.88 and ACC > 0.70, suggesting that they all have considerable ability to predict the TCM pattern type. As shown in Figure 6, using the selected features, the SVM, RF, and LR classifier achieved AUCs of 0.897, 0.880, and 0.899, respectively, indicating that the MLP model outperformed the other classifiers. As shown in Figure 7, the ACC of TCM pattern type-0, type-1, type-2, type-3, RF algorithm, MLP algorithm, SVM algorithm, and LR algorithm achieved the highest ACC of 0.85, 0.81, 0.75, and 0.71, respectively. This suggests that the performance on frequent patterns is higher than the performance on rare patterns. Notably, the ML classifiers demonstrated strong predictive power for predicting the TCM pattern type, with the MLP algorithm displaying the highest average performance. Figure 8 displays the SHAP bar plot, in which features are ranked in descending order of their contribution to model predictions (mean absolute SHAP value).

## 4. Discussion

### 4.1. Main Findings

This study investigated the use of ML to enhance ADHD management in children through parent-administered pediatric *tuina*. It involved 1005 eligible participants, selected from a large pool, based on specific criteria. The research focused on identifying key features using five-fold cross-validation, ultimately identifying seven crucial features. ML models, including MLP, SVM, LR, and RF, were employed to predict TCM patterns. The MLP model emerged as the top performer with the highest AUC and ACC. This suggests that these models are effective in capturing the complex patterns in the data, allowing for accurate classification of TCM patterns. The consistent performance across models indicates a robust framework for integrating ML with TCM, offering a promising direction for personalized ADHD treatments. By employing modern computational techniques alongside traditional TCM knowledge, this study demonstrates a practical approach to integrating ML with TCM diagnostic methods for ADHD management. The study identified several key features crucial for predicting TCM patterns in ADHD management. Features such as feature 15 (“The child is prone to allergic diseases, such as allergic rhinitis or cough variant asthma, or tends to sneeze, have a runny nose, nasal congestion, or cough under certain conditions”), 14 (“The child experiences skin itching after contact with or consumption of certain allergens”), 19 (“The child had chronic diarrhoea or eczema in early childhood”), and 37 (“The child is prone to mouth ulcers and sore throat”) showed high importance values, indicating their significant role in distinguishing different TCM patterns. These findings reflect the complexity of TCM pattern identification in ADHD management. These TCM patterns can be broadly categorized into several common basic patterns, as guided by clinical practice guidelines and expert consensus. However, within these basic patterns, each child presents with unique individual variations in terms of the specific combination of symptoms, the severity of different manifestations, and the potential presence of additional pattern features. A child’s constitution and corresponding TCM pattern may change over time due to various factors such as dietary habits, seasonal changes, geographic location, growth and development, and environmental influences. Based on our clinical research experience and practice, we have found that TCM patterns in ADHD children require monthly reassessment and pattern identification. This frequent monitoring is crucial because children’s conditions can change rapidly due to physical growth and development, changes in living environment and daily routines, seasonal transitions, dietary modifications, and response to ongoing treatment. Therefore, timely and regular pattern identification and prescription adjustments (typically monthly based on our previous clinical research) are essential to optimize treatment effectiveness, address emerging symptoms promptly, maintain therapeutic momentum, and prevent potential pattern transitions from being missed. Our ML model is designed to support this dynamic clinical process by enabling efficient and consistent pattern identification during regular follow-ups. While our framework addresses this complexity, these identified features likely correspond to physical and behavioral symptoms that align closely with ADHD characteristics, providing insights into how these traditional indicators can be used to tailor interventions. Moreover, in the analysis of feature clusters, features 7–13 exhibit a negative correlation with other features, suggesting their unique contribution to the diagnostic framework. This negative correlation indicates that, when these features are present, they may inversely affect the manifestation of other feature groups, potentially serving as distinct indicators within the diagnostic process. Further research is needed to explore the specific contexts in which these features provide significant diagnostic insights. If their relevance is confirmed, these features could be clustered together and utilized collectively in diagnostic evaluations, enhancing the precision of identifying TCM patterns in ADHD management. This approach would refine diagnostic ACC and contribute to more targeted and individualized treatment strategies.

The analysis identified strong correlations among certain questionnaire features, likely due to overlapping item content. For example, features 17 and 63 (both skin-related) show a strong positive correlation, while symptom 10 (good sleep behavior) and feature 44 (time to fall asleep) exhibit a strong negative correlation. Such correlations suggest information redundancy, indicating that not all highly correlated features are necessary for analysis. To streamline the feature set and improve model generalization, we propose consolidating feature pairs with high correlations (Pearson’s r > 0.7): 10 and 44, 17 and 63, 40 and 65, and 49 and 58. After assessing each feature’s relevance, we recommend retaining features 10, 17, 65, and 58 and removing 44, 63, 40, and 49. This refinement may enhance model accuracy and efficiency. Future studies should validate the clinical effectiveness of this streamlined questionnaire and assess its impact on diagnostic accuracy.

The research on predicting TCM patterns for ADHD management utilizing four ML models—MLP, SVM, LR, and RF—revealed challenges due to TCM pattern distribution imbalance and limited sample sizes for specific patterns. These challenges, primarily stemming from an underrepresentation of certain TCM patterns, significantly influenced model performance, as biases toward majority patterns led to skewed predictions [38,39]. Various optimization techniques tailored to each model significantly impacted their performance, with differences in strategy affecting their ability to handle the dataset’s unique challenges effectively [40,41]. To mitigate bias and improve model robustness, cross-validation techniques were employed to enhance ACC across diverse data subsets [42,43]. Despite these efforts, the scarcity of data for rarer TCM patterns remained a critical issue. Future research should expand the dataset for a more balanced representation of TCM patterns, potentially through enhanced data collection for underrepresented categories or advanced data augmentation methods [44,45]. Furthermore, refining feature selection and optimization strategies, particularly through more rigorous cross-validation methods, is recommended to boost the models’ predictive ACC and support more personalized ADHD management strategies using TCM, thereby advancing the development of equitable machine learning applications in clinical settings [46,47].

### 4.2. Implications for TCM Practitioners and ADHD Families

The model output serves as a decision support tool to assist TCM practitioners in two ways: (1) it provides an initial TCM pattern differentiation before clinical visits, helping practitioners prepare targeted treatment plans; (2) it offers quantitative evidence to support or validate practitioners’ clinical judgment during face-to-face consultations. This complements, rather than replaces, TCM practitioners’ expertise in the clinical setting. The ML model developed in this research transcends its initial application in pediatric *tuina*, demonstrating extensive applicability across a broad spectrum of TCM treatments for ADHD. This model’s robust capability to accurately identify TCM patterns significantly could enhance various modalities such as acupuncture, moxibustion, cupping, Chinese herbal prescription formulation, and tailored dietary interventions. Its advanced ability to analyze and interpret complex feature data is critical for precise TCM pattern identification, which is feasible for customizing treatment strategies like acupuncture and cupping therapy to address the unique imbalances associated with ADHD. Furthermore, this study pioneers the application of ML in pediatric *tuina* for ADHD treatment. Given the significant shortage of qualified pediatric *tuina* practitioners and ADHD’s high prevalence, this integration of ML with standardized TCM diagnostic tools ensures safer, more effective, and accessible interventions through daily home-based treatment, potentially benefiting numerous families affected by ADHD. Beyond ADHD management, the ML model shows promising potential for addressing a range of other pediatric medical conditions. Its adaptability allows for the development of targeted treatment strategies informed by deep insights derived from TCM principles. Additionally, this model serves as a helpful tool for junior TCM practitioners, offering detailed, data-driven insights that aid in disease diagnosis and treatment plan formulation.

### 4.3. Novelty and Social Impact

Our study makes significant contributions to TCM diagnosis and healthcare delivery in three key aspects: First, we innovatively integrated machine learning with the standardized TCM pattern identification questionnaire for children (released in 2023). This addresses a critical gap in pediatric TCM diagnosis, where previous pattern identification largely relied on adult questionnaires or non-standardized clinical observations. Second, our approach transforms traditional experience-based TCM pattern identification into a standardized, data-driven methodology. With the growing global demand for complementary treatments, this standardization ensures more reliable and consistent diagnosis, making TCM more accessible and applicable in modern healthcare settings. Third, by successfully integrating computational methods with standardized diagnostic tools, our work demonstrates the feasibility of combining traditional medicine with modern technology. This has substantial clinical and social impact, particularly in healthcare systems seeking to integrate traditional and modern medical approaches. Our achieved accuracy levels (>70%) are particularly meaningful given the inherent subjectivity in TCM pattern identification. This performance aligns with recent findings from similar studies in TCM constitution classification, where machine learning applications typically achieve AUC values between 0.82 and 0.95 [31]. Future research with larger datasets would be valuable for further improving identification accuracy, especially for less common patterns.

### 4.4. Strengths and Limitations

This study presents multiple strengths that significantly enhance its impact and robustness in the field of ADHD management using TCM interventions. First, five-fold cross-validation was applied in both feature extraction and model training, which helps ensure the stability and reliability of the analytical results. Second, integrating traditional Chinese medical principles with contemporary ML methods provides a practical framework for developing personalized approaches to pediatric care for children with ADHD symptoms. Third, recruiting participants from 32 provincial administrative regions across China increases the dataset’s diversity and enhances the generalizability of the findings. Fourth, the prospective design with standardized assessment and consensus-based pattern differentiation by experienced practitioners helps minimize potential biases inherent in TCM diagnosis. Finally, the use of a structured and culturally adapted parent questionnaire ensures that the clinical data collected are both comprehensive and relevant, supporting the practical translation of predictive models into clinical settings.

Despite its notable strengths, this study has several limitations that should be acknowledged. First, the data were collected exclusively from Chinese participants using a parent-reported questionnaire designed for local TCM constitution assessment, which may limit the generalizability of the findings to other populations or cultural settings. To apply this approach in different cultural contexts, the questionnaire would require cultural adaptation, including language translation and modification of TCM concepts to align with local healthcare practices. The validity of TCM pattern differentiation criteria may vary across cultures due to differences in lifestyle, diet, and environmental factors. Second, the reliance on parent-reported data introduces inherent subjectivity and variability, potentially affecting data quality and, consequently, the accuracy and robustness of the ML models in identifying TCM patterns and assessing ADHD symptoms. Parent reports may be influenced by recall bias, varying levels of observation, and different interpretation standards of children’s behaviors. Third, the uneven distribution of TCM patterns and the relatively small sample size for certain subgroups may may limit detection of rare but clinically meaningful effects. Finally, although the models demonstrated promising predictive performance, their accuracy may not yet meet the high standards required for clinical application. Future research with larger, more diverse, and multi-center cohorts, as well as external validation in different populations, will be essential to enhance the robustness and applicability of our approach. While our current study utilized a rigorous three-way data split approach, future research should focus on validating the model using independent datasets from different hospitals and regions. This external validation would help establish model generalizability across different clinical settings and patient populations. Furthermore, we plan to conduct RCTs to compare the clinical efficacy and feasibility between prescriptions generated by our model and those prescribed by TCM practitioners through traditional pattern differentiation. Such clinical trials will provide crucial evidence for the practical application value of our ML-assisted TCM diagnostic model.

In the past few years, the COVID-19 pandemic saw the widespread use of TCM as a supportive treatment modality, particularly in regions like China, where TCM was integrated into national COVID-19 treatment guidelines. Real-world evidence from electronic health records and patient registries could provide valuable insights into the efficacy and safety of TCM interventions [48]. However, analyzing such data presents unique challenges due to the complexity of TCM formulations, individualized treatment principles, and the need for advanced natural language processing techniques to extract meaningful patterns from unstructured clinical notes. Machine learning methods could be adapted to identify TCM treatment patterns, predict patient responses, and integrate multi-omics data to understand mechanistic pathways. Future research should explore how big data analytics can bridge traditional medicine with modern healthcare systems, especially in pandemic preparedness and response.

## 5. Conclusions

This study developed an ML-based approach for enhancing the management of ADHD in children using pediatric *tuina*, as informed by a parent-reported questionnaire. Seven key features (relating to allergic conditions, emotional characteristics, and gastrointestinal symptoms) were selected for conducting TCM pattern identification, developing personalized treatment strategies, and identifying the most suitable ML models for these tasks. The use of multiple ML models, including MLP, SVM, LR, and RF, demonstrated strong predictive capabilities, with the MLP model achieving the highest AUC and ACC. Future research should focus on expanding data collection for underrepresented TCM patterns, validating the model across diverse populations, and evaluating the clinical effectiveness of ML-guided treatment strategies.

## Figures and Tables

**Table 1:** Characteristics of children labeled as different TCM patterns.

	Participants Labeled as 0 ^d^ (*N* = 471)	Participants Labeled as 1 ^e^ (*N* = 212)	Participants Labeled as 2 ^f^ (*N* = 239)	Participants Labeled as 3 ^g^ (*N* = 83)	*p*-Value
Children					
Age, median (IQR)	10.0 (3.0)	10.0 (2.0)	10.0 (2.0)	10.0 (2.0)	0.087
Gender, *n* (%)					0.366
Male	392 (83.2)	169 (79.7)	204 (85.4)	66 (79.5)	
Female	79 (16.8)	43 (20.3)	35 (14.6)	17 (20.5)	
BMI, median (IQR)	16.0 (4.7)	16.0 (4.2)	16.7 (4.7)	16.3 (4.0)	0.234
Family history of allergy, *n* (%)					0.000
Yes	245 (52.0)	67 (31.6)	50 (20.9)	21 (25.3)	
No	226 (48.0)	145 (68.4)	189 (79.1)	62 (74.7)	
Frequency of respiratory infections per year, *n* (%)					0.000
Always ^a^	82 (17.4)	23 (10.8)	22 (9.2)	12 (14.5)	
Sometime ^b^	262 (55.6)	115 (54.2)	115 (48.1)	52 (62.7)	
Rarely ^c^	127 (27.0)	74 (34.9)	102 (42.7)	19 (22.9)	
Parents					
Education level, *n* (%)					0.178
Primary school or below	0 (0)	1 (0.5)	0 (0)	0 (0)	
Middle school	19 (4.0)	13 (6.1)	15 (6.3)	5 (6.0)	
Junior college	82 (17.4)	45 (21.2)	52 (21.8)	23 (27.7)	
College or above	370 (78.6)	153 (72.2)	172 (72.0)	55 (66.3)	
Family income, *n* (%)					1.000
Below CNY 4999	4 (0.8)	5 (2.4)	10 (4.2)	3 (3.6)	
CNY 5000–9999	78 (16.6)	42 (19.8)	39 (16.3)	15 (18.1)	
CNY 10,000–29,999	227 (48.2)	104 (49.1)	122 (51.0)	47 (56.6)	
CNY 30,000–49,999	103 (21.9)	33 (15.6)	43 (18.0)	14 (16.9)	
CNY 50,000 or above	59 (12.5)	28 (13.2)	25 (10.5)	4 (4.8)	

Remarks: a: frequency of respiratory infections in children per year (always): ages 1–3: ≥7 times/year, ages 4–6: ≥6 times/year, ages 7–12: ≥5 times/year; b: frequency of respiratory infections in children per year (sometime): ages 1–3: 2–6 times/year, ages 4–6: 2–5 times/year, ages 7–12: 2–4 times/year; c: frequency of respiratory infections in children per year (rarely): <2 times/year; d: pattern of dual deficiency of the lung–spleen; e: pattern of liver depression and spleen deficiency; f: pattern of liver ascendant hyperactivity and spleen deficiency; g: other patterns; CNY: Chinese Yuan.

## Data Availability

The correlation analysis data supporting this study are provided in Supplementary Materials. The original de-identified clinical data is available on request from the corresponding author with appropriate justification.

## Supplementary Materials

The following supporting information can be downloaded at: https://www.mdpi.com/article/10.3390/bioengineering12101012/s1, S1: Traditional Chinese Medicine Child Constitution Assessment (For Parents); S2: TCM Pattern Identification Approaches and Corresponding Pediatric *Tuina* Prescriptions for ADHD (based on our previous research experience. The symptoms are according to the Traditional Chinese Medicine Children’s Constitution Scale); S3a: All features’ correlation; S3b: All features’ correlation pval; S3c: Selected features’ correlation; S3d: Selected features’ correlation pval.
