# Supplementary material for: Feature Selection and Prediction of Pediatric Tuina in Attention Deficit/Hyperactivity Disorder Management: A Machine Learning Approach Based on Parent-Reported Children’s Constitution
Source: Bioengineering (Basel). 2025 Sep 23;12(10):1012. doi: 10.3390/bioengineering12101012 (PMC12561778; doi:10.3390/bioengineering12101012)
Supplement: Supplementary file 1 [file bioengineering-12-01012-s001.zip › S1.docx]

**Supplementary S1. Traditional Chinese Medicine Child Constitution Assessment (For Parents)**

| 1. Age |  | | | | | |
| --- | --- | --- | --- | --- | --- | --- |
| 1. How often does your child get respiratory infections (such as colds, bronchitis, pneumonia) annually? | - Ages 1-3: ≥7 times/year (1) - Ages 1-3: 2-6 times/year (2) - Ages 4-6: ≥6 times/year (3) - Ages 4-6: 2-5 times/year (4) - Ages 7-12: ≥5 times/year (5) - Ages 7-12: 2-4 times/year (6) - <2 times/year (7) | | | | | |
| 1. Does your child rarely get sick? | Yes (1) | | | No (0) | | |
| 1. Does your child have a family history of allergic diseases? | Yes (1) | | | No (0) | | |
| 1. Does your child have a history of asthma? | Yes (1) | | | No (0) | | |
| 1. Was your child born with a low birth weight? | Yes (1) | | | No (0) | | |
| 1. The child is energetic. | Always (5) | Often (4) | Sometimes (3) | | Occasionally (2) | Never (1) |
| 1. The child has a loud voice (or cries loudly). | Always (5) | Often (4) | Sometimes (3) | | Occasionally (2) | Never (1) |
| 1. The child has a good appetite and eats a normal amount. | Always (5) | Often (4) | Sometimes (3) | | Occasionally (2) | Never (1) |
| 1. The child falls asleep quickly and sleeps soundly. | Always (5) | Often (4) | Sometimes (3) | | Occasionally (2) | Never (1) |
| 1. The child has normal stool consistency and defecates 1-2 times a day. | Always (5) | Often (4) | Sometimes (3) | | Occasionally (2) | Never (1) |
| 1. The child has a cheerful personality. | Always (5) | Often (4) | Sometimes (3) | | Occasionally (2) | Never (1) |
| 1. The child recovers quickly after being sick. | Always (5) | Often (4) | Sometimes (3) | | Occasionally (2) | Never (1) |
| 1. The child experiences itching after contact with or consuming allergens (such as certain foods, pollen, dust, pets, etc.). | Always (5) | Often (4) | Sometimes (3) | | Occasionally (2) | Never (1) |
| 1. The child is prone to allergic diseases, such as allergic rhinitis or cough-variant asthma, or tends to sneeze, have a runny nose, nasal congestion, or cough in situations like seasonal changes, temperature fluctuations, or exposure to pollen or furry animals, or in environments with potential allergens like renovations. | Always (5) | Often (4) | Sometimes (3) | | Occasionally (2) | Never (1) |
| 1. The child easily experiences stomach pain or diarrhea after eating certain foods. | Always (5) | Often (4) | Sometimes (3) | | Occasionally (2) | Never (1) |
| 1. The child easily develops eczema or hives. | Always (5) | Often (4) | Sometimes (3) | | Occasionally (2) | Never (1) |
| 1. The child likes to rub their nose, rub their eyes, or blink frequently. | Always (5) | Often (4) | Sometimes (3) | | Occasionally (2) | Never (1) |
| 1. The child had chronic diarrhea or eczema when they were younger. | Always (5) | Often (4) | Sometimes (3) | | Occasionally (2) | Never (1) |
| 1. The child has a soft voice (or cries softly and timidly). | Always (5) | Often (4) | Sometimes (3) | | Occasionally (2) | Never (1) |
| 1. The child’s stool is unformed or contains undigested food. | Always (5) | Often (4) | Sometimes (3) | | Occasionally (2) | Never (1) |
| 1. The child sweats easily after activity. | Always (5) | Often (4) | Sometimes (3) | | Occasionally (2) | Never (1) |
| 1. The child prefers quiet activities and doesn’t like outdoor activities. | Always (5) | Often (4) | Sometimes (3) | | Occasionally (2) | Never (1) |
| 1. The child is timid and speaks little. | Always (5) | Often (4) | Sometimes (3) | | Occasionally (2) | Never (1) |
| 1. The child has a bloated stomach. | Always (5) | Often (4) | Sometimes (3) | | Occasionally (2) | Never (1) |
| 1. The child has a poor appetite. | Always (5) | Often (4) | Sometimes (3) | | Occasionally (2) | Never (1) |
| 1. The child feels discomfort, such as stomach pain or diarrhea, after eating cold food. | Always (5) | Often (4) | Sometimes (3) | | Occasionally (2) | Never (1) |
| 1. The child is often sleepy and drowsy. | Always (5) | Often (4) | Sometimes (3) | | Occasionally (2) | Never (1) |
| 1. The child is afraid of the cold. | Always (5) | Often (4) | Sometimes (3) | | Occasionally (2) | Never (1) |
| 1. The child has cold hands and feet. | Always (5) | Often (4) | Sometimes (3) | | Occasionally (2) | Never (1) |
| 1. The child takes a long time to fall asleep or wakes up easily. | Always (5) | Often (4) | Sometimes (3) | | Occasionally (2) | Never (1) |
| 1. The child has dry stool. | Always (5) | Often (4) | Sometimes (3) | | Occasionally (2) | Never (1) |
| 1. The child has warm palms and feet. | Always (5) | Often (4) | Sometimes (3) | | Occasionally (2) | Never (1) |
| 1. The child sweats easily while sleeping. | Always (5) | Often (4) | Sometimes (3) | | Occasionally (2) | Never (1) |
| 1. The child has a quick temper. | Always (5) | Often (4) | Sometimes (3) | | Occasionally (2) | Never (1) |
| 1. The child has dry or itchy skin. | Always (5) | Often (4) | Sometimes (3) | | Occasionally (2) | Never (1) |
| 1. The child easily develops mouth ulcers or sore throats. | Always (5) | Often (4) | Sometimes (3) | | Occasionally (2) | Never (1) |
| 1. The child is highly energetic and very active. | Always (5) | Often (4) | Sometimes (3) | | Occasionally (2) | Never (1) |
| 1. The child has a large appetite and gets hungry easily. | Always (5) | Often (4) | Sometimes (3) | | Occasionally (2) | Never (1) |
| 1. The child doesn’t sleep soundly and tosses and turns frequently. | Always (5) | Often (4) | Sometimes (3) | | Occasionally (2) | Never (1) |
| 1. The child’s stool smells foul. | Always (5) | Often (4) | Sometimes (3) | | Occasionally (2) | Never (1) |
| 1. The child is afraid of heat and sweats a lot after activity. | Always (5) | Often (4) | Sometimes (3) | | Occasionally (2) | Never (1) |
| 1. The child has a lot of eye discharge upon waking. | Always (5) | Often (4) | Sometimes (3) | | Occasionally (2) | Never (1) |
| 1. The child takes a long time to fall asleep. | Always (5) | Often (4) | Sometimes (3) | | Occasionally (2) | Never (1) |
| 1. The child is sensitive and thoughtful, caring a lot about others’ opinions. | Always (5) | Often (4) | Sometimes (3) | | Occasionally (2) | Never (1) |
| 1. The child tends to feel melancholic and sighs often. | Always (5) | Often (4) | Sometimes (3) | | Occasionally (2) | Never (1) |
| 1. The child easily feels anxious and overthinks things. | Always (5) | Often (4) | Sometimes (3) | | Occasionally (2) | Never (1) |
| 1. The child remains in a low mood for a long time after setbacks. | Always (5) | Often (4) | Sometimes (3) | | Occasionally (2) | Never (1) |
| 1. The child often experiences hiccups or nausea and dry retching. | Always (5) | Often (4) | Sometimes (3) | | Occasionally (2) | Never (1) |
| 1. The child feels like there’s something stuck in their throat. | Always (5) | Often (4) | Sometimes (3) | | Occasionally (2) | Never (1) |
| 1. The child experiences unexplained headaches. | Always (5) | Often (4) | Sometimes (3) | | Occasionally (2) | Never (1) |
| 1. The child is slow to adapt to group life after starting school. | Always (5) | Often (4) | Sometimes (3) | | Occasionally (2) | Never (1) |
| 1. The child tires easily and lacks energy. | Always (5) | Often (4) | Sometimes (3) | | Occasionally (2) | Never (1) |
| 1. The child doesn’t like to drink water. | Always (5) | Often (4) | Sometimes (3) | | Occasionally (2) | Never (1) |
| 1. The child’s stool is unformed. | Always (5) | Often (4) | Sometimes (3) | | Occasionally (2) | Never (1) |
| 1. The child sweats and feels sticky. | Always (5) | Often (4) | Sometimes (3) | | Occasionally (2) | Never (1) |
| 1. The child tends to procrastinate and has a slow-paced personality. | Always (5) | Often (4) | Sometimes (3) | | Occasionally (2) | Never (1) |
| 1. The child often experiences hiccups or nausea and dry retching. | Always (5) | Often (4) | Sometimes (3) | | Occasionally (2) | Never (1) |
| 1. The child feels like there is phlegm in their throat. | Always (5) | Often (4) | Sometimes (3) | | Occasionally (2) | Never (1) |
| 1. The child easily has a lot of phlegm when coughing. | Always (5) | Often (4) | Sometimes (3) | | Occasionally (2) | Never (1) |
| 1. The child often cries or wakes up startled at night. | Always (5) | Often (4) | Sometimes (3) | | Occasionally (2) | Never (1) |
| 1. The child’s stool sticks to the toilet bowl and is difficult to flush. | Always (5) | Often (4) | Sometimes (3) | | Occasionally (2) | Never (1) |
| 1. The child easily develops eczema. | Always (5) | Often (4) | Sometimes (3) | | Occasionally (2) | Never (1) |
| 1. The child has bad breath. | Always (5) | Often (4) | Sometimes (3) | | Occasionally (2) | Never (1) |
| 1. The child doesn’t sleep soundly and tosses and turns or likes to sleep on their stomach. | Always (5) | Often (4) | Sometimes (3) | | Occasionally (2) | Never (1) |
| 1. The child grinds their teeth while sleeping. | Always (5) | Often (4) | Sometimes (3) | | Occasionally (2) | Never (1) |
| 1. The child easily experiences stomach pain or bloating. | Always (5) | Often (4) | Sometimes (3) | | Occasionally (2) | Never (1) |
| 1. The child’s burps often have a sour odor. | Always (5) | Often (4) | Sometimes (3) | | Occasionally (2) | Never (1) |
| 1. The child tends to overeat and has indigestion. | Always (5) | Often (4) | Sometimes (3) | | Occasionally (2) | Never (1) |
| 1. The child’s stomach easily feels bloated after meals. | Always (5) | Often (4) | Sometimes (3) | | Occasionally (2) | Never (1) |
| 1. The child wets the bed while sleeping. | Always (5) | Often (4) | Sometimes (3) | | Occasionally (2) | Never (1) |

**Appendix 2**

| **Feature No.** | **Train**  **(AUC)** | **Validation**  **(AUC)** | **Train**  **(Accuracy)** | **Validation**  **(Accuracy)** |
| --- | --- | --- | --- | --- |
| 1 | 0.736748 | 0.706753 | 0.51087 | 0.5 |
| 2 | 0.802138 | 0.774906 | 0.60559 | 0.53125 |
| 3 | 0.836442 | 0.801742 | 0.619565 | 0.56875 |
| 4 | 0.863217 | 0.813105 | 0.673913 | 0.60625 |
| 5 | 0.882572 | 0.831074 | 0.695652 | 0.61875 |
| 6 | 0.891614 | 0.841763 | 0.690994 | 0.64375 |
| 7 | 0.899375 | 0.85669 | 0.697205 | 0.65625 |
| 8 | 0.906262 | 0.856713 | 0.725155 | 0.675 |
| 9 | 0.91046 | 0.859489 | 0.717391 | 0.6625 |
| 10 | 0.914969 | 0.862772 | 0.725155 | 0.675 |
| 11 | 0.917708 | 0.858782 | 0.737578 | 0.66875 |
| 12 | 0.920245 | 0.857322 | 0.737578 | 0.66875 |
| 13 | 0.921423 | 0.853173 | 0.740683 | 0.65625 |
| 14 | 0.923704 | 0.852417 | 0.73913 | 0.675 |
| 15 | 0.925784 | 0.847235 | 0.743789 | 0.6625 |
| 16 | 0.926875 | 0.849404 | 0.748447 | 0.66875 |
| 17 | 0.927982 | 0.848358 | 0.751553 | 0.66875 |
| 18 | 0.929046 | 0.849262 | 0.746894 | 0.65625 |
| 19 | 0.931451 | 0.853027 | 0.748447 | 0.64375 |
| 20 | 0.932104 | 0.853712 | 0.754658 | 0.64375 |
| 21 | 0.932315 | 0.854594 | 0.753106 | 0.64375 |
| 22 | 0.932653 | 0.853139 | 0.762422 | 0.625 |
| 23 | 0.933085 | 0.851992 | 0.76087 | 0.6375 |
| 24 | 0.933706 | 0.851558 | 0.767081 | 0.63125 |
| 25 | 0.934571 | 0.849958 | 0.763975 | 0.6375 |
| 26 | 0.934912 | 0.848084 | 0.762422 | 0.63125 |
| 27 | 0.934845 | 0.850325 | 0.763975 | 0.625 |
| 28 | 0.935025 | 0.848692 | 0.770186 | 0.6375 |
| 29 | 0.936232 | 0.845221 | 0.774845 | 0.61875 |
| 30 | 0.936719 | 0.84506 | 0.771739 | 0.625 |
| 31 | 0.936613 | 0.845024 | 0.774845 | 0.6125 |
| 32 | 0.936419 | 0.845816 | 0.771739 | 0.6125 |
| 33 | 0.936616 | 0.841973 | 0.776398 | 0.61875 |
| 34 | 0.937327 | 0.841349 | 0.770186 | 0.6125 |
| 35 | 0.93746 | 0.840582 | 0.770186 | 0.60625 |
| 36 | 0.937685 | 0.840002 | 0.771739 | 0.60625 |
| 37 | 0.939365 | 0.83539 | 0.781056 | 0.6125 |
| 38 | 0.939954 | 0.830616 | 0.776398 | 0.59375 |
| 39 | 0.941036 | 0.831071 | 0.785714 | 0.625 |
| 40 | 0.94164 | 0.828316 | 0.785714 | 0.6125 |
| 41 | 0.942297 | 0.821404 | 0.785714 | 0.6 |
| 42 | 0.942414 | 0.821782 | 0.784161 | 0.5875 |
| 43 | 0.943002 | 0.822029 | 0.791925 | 0.59375 |
| 44 | 0.944459 | 0.827124 | 0.799689 | 0.60625 |
| 45 | 0.945123 | 0.826184 | 0.802795 | 0.59375 |
| 46 | 0.94626 | 0.824162 | 0.802795 | 0.6 |
| 47 | 0.946279 | 0.825272 | 0.804348 | 0.625 |
| 48 | 0.946612 | 0.824297 | 0.802795 | 0.625 |
| 49 | 0.946666 | 0.824897 | 0.805901 | 0.60625 |
| 50 | 0.947316 | 0.828489 | 0.812112 | 0.61875 |
| 51 | 0.948319 | 0.828512 | 0.807453 | 0.6375 |
| 52 | 0.948252 | 0.83055 | 0.807453 | 0.63125 |
| 53 | 0.948667 | 0.828829 | 0.807453 | 0.6375 |
| 54 | 0.949393 | 0.827457 | 0.810559 | 0.65 |
| 55 | 0.949034 | 0.827132 | 0.810559 | 0.6375 |
| 56 | 0.949721 | 0.827667 | 0.809006 | 0.65 |
| 57 | 0.949716 | 0.825336 | 0.810559 | 0.63125 |
| 58 | 0.949799 | 0.827175 | 0.813665 | 0.625 |
| 59 | 0.949875 | 0.826941 | 0.812112 | 0.625 |
| 60 | 0.950552 | 0.828165 | 0.815217 | 0.61875 |
| 61 | 0.950993 | 0.831804 | 0.819876 | 0.61875 |
| 62 | 0.951563 | 0.832289 | 0.824534 | 0.61875 |
| 63 | 0.953162 | 0.826138 | 0.829193 | 0.625 |
| 64 | 0.953591 | 0.823477 | 0.830745 | 0.60625 |
| 65 | 0.953894 | 0.819386 | 0.826087 | 0.625 |
| 66 | 0.95421 | 0.820682 | 0.824534 | 0.625 |
| 67 | 0.954404 | 0.819623 | 0.819876 | 0.60625 |
| 68 | 0.954841 | 0.822049 | 0.824534 | 0.6125 |
| 69 | 0.955767 | 0.823711 | 0.830745 | 0.6 |
