# Supplementary material for: Feature Selection and Prediction of Pediatric Tuina in Attention Deficit/Hyperactivity Disorder Management: A Machine Learning Approach Based on Parent-Reported Children’s Constitution
Source: Bioengineering (Basel). 2025 Sep 23;12(10):1012. doi: 10.3390/bioengineering12101012 (PMC12561778; doi:10.3390/bioengineering12101012)
Supplement: Supplementary file 1 [file bioengineering-12-01012-s001.zip › S2.docx]

**Supplementary S2. TCM Pattern Identification Approaches and Corresponding Pediatric *Tuina* Prescriptions for ADHD (Based on our previous research experience. The symptoms are according with the Traditional Chinese Medicine Children’s Constitution Scale)**

| **證型**  **TCM Pattern** |  | **相關聯兒童症狀**  **Related Children's Symptoms** |  | **小兒推拿處方**  **Pediatric *Tuina* Prescription** |
| --- | --- | --- | --- | --- |
| 肺脾氣虛  Dual deficiency of the lung-spleen pattern |  | 易過敏(Allergic predisposition)、易腹痛腹瀉(Prone to abdominal pain and diarrhea)、易起濕疹/蕁麻疹(Susceptible to eczema/urticaria)、喜歡揉鼻子、揉眼睛或眨眼(Habitual rhinotillexis and eye rubbing/frequent blinking)、活動後易出汗(Hyperhidrosis during physical activity)、喜歡安靜(Preference for quiet environments)、不愛戶外活動(Aversion to outdoor activities)、膽子小(Timidity/anxiety tendencies)、說話少(Reduced verbal communication/reticence)、肚子脹(Abdominal distention/bloating)、食欲差(Poor appetite/decreased food intake)、多眠易困(Hypersomnia/somnolence)、怕冷(Cold intolerance)、手腳涼(Acral coldness/cold extremities)、咳嗽時容易痰多(Productive cough with excessive sputum)、容易勞累(Fatigue proneness)、沒精神(Lethargy/asthenia)、不喜歡喝水(Poor fluid intake/hypohydration tendency)、大便不成形(Loose stools/unformed feces)、做事拖拖拉拉/性子慢(Psychomotor retardation/bradykinesia in task execution) |  | 拿五經 (Rubbing Wu Jing)  捏脊 (Spine Pinching)  按揉足三裏 (Pressing Zusanli)  摩腹 (Abdominal Rubbing)  按揉三陰交 (Pressing Sanyinjiao) |
| 肝鬱脾虛  Liver depression and spleen deficiency pattern |  | 心思細膩/敏感/很在乎別人的看法(Heightened sensitivity/emotional delicacy/excessive concern for others' opinions)、容易悶悶不樂/唉聲歎氣(Prone to melancholy/frequent sighing)、容易焦慮，想事太多(Anxiety proneness/overthinking)、受挫後情緒低落持續較久(Prolonged emotional recovery from setbacks)、易打嗝或噁心幹嘔(Frequent eructation or nausea with dry heaving)、喉間有異物感(Globus sensation)、喉嚨有痰(Pharyngeal mucus accumulation)、無明顯原因的頭痛(Idiopathic headache)、入學後適應集體生活慢(Delayed adaptation to collective/school life)、容易勞累/沒精神(Fatigue proneness/asthenia)、不喜歡喝水(Poor fluid intake/hypohydration tendency)、大便不成形(Loose stools/unformed feces)、做事拖拖拉拉、性子慢(Psychomotor retardation/bradykinesia in task execution)、肚子脹(Abdominal distention/bloating)、食欲差/吃涼的食物會感到不適如腹痛/腹瀉(Poor appetite/gastrointestinal sensitivity to cold food manifesting as abdominal pain/diarrhea)、多眠易困(Hypersomnia/somnolence) |  | 拿五經 (Rubbing Wu Jing)  開天門 (Pushing Tianmen)  推坎宮 (Pushing Kangong)  補脾經 (Tonifying Pijing)  摩腹 (Abdominal Rubbing)  按揉足三裏 (Pressing Zusanli) |
| 肝旺脾虛  Liver ascendant hyperactivity and spleen deficiency pattern |  | 精力充沛(Hyperactivity/abundant energy)、聲音洪亮/哭聲洪亮(Sonorous voice/loud crying)、大便氣味臭(Malodorous stools)、怕熱(Heat intolerance)、活動後出汗多(Exercise-induced hyperhidrosis)、睡眠不踏實/來回翻滾(Restless sleep/frequent positional changes)、睡覺磨牙(Sleep bruxism)、脾氣急躁(Irritability/quick temper)、晨起眼屎多(Excessive matutinal eye discharge)、口氣重(Halitosis)、肚子脹(Abdominal distention/bloating)、食欲差(Poor appetite/decreased food intake)、吃涼的食物會感到不適如腹痛/腹瀉(Gastrointestinal sensitivity to cold food manifesting as abdominal pain/diarrhea)、多眠易困(Hypersomnia/somnolence)、大便不成形(Loose stools/unformed feces) |  | 拿五經 (Rubbing Wu Jing)  清肝經 (Clearing Ganjing)  補脾經 (Tonifying Pijing)  按揉足三裏 (Pressing Zusanli)  摩腹 (Abdominal Rubbing) |
| 心肝火旺  Effulgent heart-liver fire pattern |  | 精力充沛(Hyperactivity/abundant energy)、聲音洪亮/哭聲洪亮(Sonorous voice/loud crying)、大便氣味臭(Malodorous stools)、怕熱(Heat intolerance)、活動後出汗多(Exercise-induced hyperhidrosis)、睡眠不踏實/來回翻滾(Restless sleep/frequent positional changes)、睡覺磨牙(Sleep bruxism)、易起口瘡/喉嚨痛(Recurrent aphthous stomatitis/pharyngitis)、脾氣急躁(Irritability/quick temper)、晨起眼屎多(Excessive matutinal eye discharge)、口氣重(Halitosis)、出汗黏(Sticky/viscous sweating) |  | 清心經 (Clearing Xinjing)  清肝經 (Clearing Ganjing)  清大腸 (Clearing Dachangjing)  拿五經 (Rubbing Wu Jing)  按揉曲池 (Pressing Quchi) |
| 痰火內擾  Phlegm-fire harassing the heart pattern |  | 精力充沛(Hyperactivity/abundant energy)、聲音洪亮/哭聲洪亮(Sonorous voice/loud crying)、大便氣味臭(Malodorous stools)、怕熱(Heat intolerance)、活動後出汗多(Exercise-induced hyperhidrosis)、睡眠不踏實/來回翻滾(Restless sleep/frequent positional changes)、睡覺磨牙(Sleep bruxism)、易起口瘡/喉嚨痛(Recurrent aphthous stomatitis/pharyngitis)、脾氣急躁(Irritability/quick temper)、晨起眼屎多(Excessive matutinal eye discharge)、口氣重(Halitosis)、咳嗽時容易痰多(Productive cough with excessive sputum)、打嗝易有酸臭味(Foul/acidic eructation)、有進食過多、積食情況(Overeating tendency/food stagnation)、飯後容易肚子脹(Postprandial abdominal distention)、易起濕疹(Susceptible to eczema)、大便黏便盆/不易沖刷乾淨(Sticky stools/difficult to flush clean)、出汗黏(Sticky/viscous sweating) |  | 拿五經 (Rubbing Wu Jing)  清肝經 (Clearing Ganjing)  清心經 (Clearing Xinjing)  清大腸 (Clearing Dachangjing)  按揉足三裏 (Pressing Zusanli) |
| 肝腎陰虛  Liver-kidney yin deficiency pattern |  | 睡覺尿床(Nocturnal enuresis)、晚上睡覺容易哭或驚醒(Nocturnal crying/night terrors)、手腳心熱(Palmar and plantar hyperthermia)、睡覺時易出汗(Night sweating)、大便乾燥(Dry/hard stools)、皮膚乾燥或易瘙癢(Xerosis cutis/pruritus)、易起口瘡/喉嚨痛(Recurrent aphthous stomatitis/pharyngitis)、出汗黏(Sticky/viscous sweating) |  | 捏脊 (Pinching Spine)  拿五經 (Rubbing Wu Jing)  清大腸 (Clearing Dachangjing)  按揉三陰交 (Pressing Sanyinjiao)揉腎頂 (Rubbing Shending) |
